# Compositions, Formation Mechanism, and Neuroprotective Effect of Compound Precipitation from the Traditional Chinese Prescription Huang-Lian-Jie-Du-Tang

**DOI:** 10.3390/molecules21081094

**Published:** 2016-08-19

**Authors:** Chenze Zhang, Rui Zhao, Wenqiang Yan, Hui Wang, Menglu Jia, Nailiang Zhu, Yindi Zhu, Yuzhong Zhang, Penglong Wang, Haimin Lei

**Affiliations:** 1 School of Chinese Pharmacy, Beijing University of Chinese Medicine, Beijing 100102, China; zcz920418@163.com (C.Z.); zr1012@bucm.edu.cn (R.Z.); ywq3226925@163.com (W.Y.); 15652387323@163.com (H.W.); 15001290933@163.com (M.J.); 2 Institute of Medicinal Plant Development, Peking Union Medical College and Chinese Academy of Medical Sciences, Beijing 100193, China; nlzhu@implad.ac.cn (N.Z.); zhuyindi314@sina.com (Y.Z.); 3 Department of Pathology, Beijing University of Chinese Medicine, Beijing 100102, China; zyz100102@126.com

**Keywords:** compounds in the form of precipitation, Huang-Lian-Jie-Du-Tang, material foundation, baicalin–berberine complex, formation mechanism, neuroprotective effect

## Abstract

Compounds in the form of precipitation (CFP) are universally formed during the decocting of Chinese prescriptions, such as Huang-Lian-Jie-Du-Tang (HLJDT). The formation rate of HLJDT CFP even reached 2.63% ± 0.20%. The identification by liquid chromatography mass spectrometry (LC-MS^n^) proved that the main chemical substances of HLJDT CFP are baicalin and berberine, which is coincident with the theory that the CFP might derive from interaction between acidic and basic compounds. To investigate the formation mechanism of HLJDT CFP, baicalin and berberine were selected to synthesize a simulated precipitation and then the baicalin–berberine complex was obtained. Results indicated that the melting point of the complex interposed between baicalin and berberine, and the UV absorption, was different from the mother material. In addition, ^1^H-NMR integral and high-resolution mass spectroscopy (HR-MS) can validate that the binding ratio was 1:1. Compared with baicalin, the chemical shifts of H and C on glucuronide had undergone significant changes by ^1^H-, ^13^C-NMR, which proved that electron transfer occurred between the carboxylic proton and the lone pair of electrons on the N atom. Both HLJDT CFP and the baicalin–berberine complex showed protective effects against cobalt chloride-induced neurotoxicity in differentiated PC12 cells. It is a novel idea, studying the material foundation of CFP in Chinese prescriptions.

## 1. Introduction

Compounds in the form of precipitation (CFP) are universally formed during the decocting of many Chinese medicinal prescriptions, such as Huang-Lian-Jie-Du-Tang (HLJDT), Ma-Xing-Shi-Gan-Tang, etc. [1,2,3]. The CFP are often abandoned with the herb residue before drinking, or in the process of separation and extraction in pharmaceutical companies. Ignoring the CFP will cause waste of the active substance, and also lead to lower drug efficacy. However, recent studies have focused mostly on the influence of forming CFP on chemical composition content in the water decoction [4,5]. The systematic study on chemical compositions and the formation mechanism of the CFP will certainly be beneficial on evaluating its medicinal value and raising its utilization. 

Huang-Lian-Jie-Du-Tang (HLJDT), one commonly used Chinese medicinal prescription, which is composed of four commonly used medicinal herbs, namely Coptidis Rhizoma, Radix Scutellariae, Phellodendri Cortex, and Gardeniae Fructus, in 3:2:2:3 proportions, is historically employed for clearing heat and detoxifying [6,7]. It is widely used for alleviating ischemic brain injury, gastrointestinal disorders, inflammation, and cardiovascular diseases [8,9,10,11,12,13,14]. In our research, the formation rate of HLJDT CFP even reached 2.63% ± 0.21% during water decocting. The formation of HLJDT CFP may cause problems in quality analysis of traditional chinese medicine (TCM) manufacturing process and lead to lower efficacy. Thus, it is necessary to identify the chemical compositions and verify the neuroprotective effect of HLJDT CFP. 

According to reference, the CFP might derive from the interaction between acidic components and basic components of HLJDT decoction [15,16,17]. This means that electron transfer might occur between the carboxyl proton on acidic component and the lone pair of electrons on basic component N atoms. In other words, a generalized acid-base reaction happened and the complex precipitation was generated. To further verify the possible formation mechanism of HLJDT CFP, the main acid-base compositions, baicalin and berberine, were identified by a LC-MS^n^ method and selected to synthesize a simulated precipitation. The baicalin–berberine complex was obtained by crystallization [18]. Due to the weak bonding of complexes, normally chromatographic methods may break the binding and fail to get the structural characteristics of complexes. Nuclear magnetic resonance (NMR) and ultraviolet (UV) absorption spectrum technologies, by detecting chemical shifts and the change of the ultraviolet absorption, are widely used in the analysis of the weak interaction between substances [19,20]. The change of ultraviolet absorption can prove that the conjugate structure have been changed. The different chemical shifts detected by superconducting high-resolution NMR can explain the variation of chemical structure. In this research, melting point test, UV absorption spectra, superconducting high-resolution NMR and high-resolution mass spectroscopy (HR-MS) were used to identify the complexation of the baicalin–berberine complex. CoCl_2_-induced neurotoxicity in differentiated PC12 cells is commonly used to screen new candidates for the intervention of ischemic brain injury [21,22]. Herein, the neuroprotective activities which were coincident with the HLJDT decoction, were evaluated on CoCl_2_-induced damage in differentiated PC12 cells by thiazolyl blue (MTT) assay, Giemsa staining, and AO/EB staining [23,24,25].

## 2. Results

### 2.1. Preparation of HLJDT CFP

**Figure 1:**
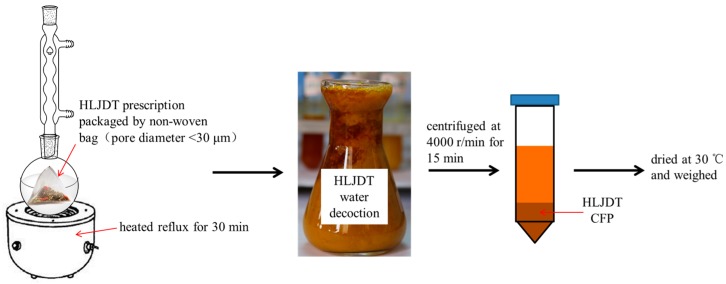
Preparation process of HLJDT CFP.

As shown in Figure 1, HLJDT prescription was packaged in a non-woven bag (pore diameter <30 μm) to avoid the interference of herb residue and heated reflux with eight times the amount of water for 30 min. HLJDT CFP was obtained by centrifugation and then dried at 30 °C. The result showed that the HLJDT CFP formation rate of three different batches is 2.63% ± 0.20% (Table 1). The formation process of HLJDT CFP is stable and controllable.

### 2.2. LC-MS^n^ Analysis of the Constituents of HLJDT CFP

**Figure 2:**
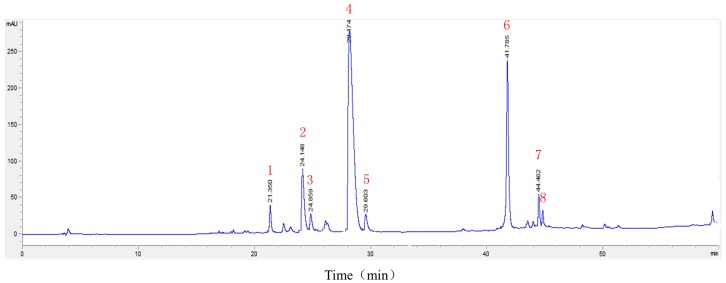
The chromatogram of HLJDT CFP. The numbers 1–8 represent eight major chemical constituents.

An LC-ESI-MS^n^ method was developed to identify the main constituents of the HLJDT CFP. electrospray ionization (ESI) mass spectra were acquired both in positive-ion and negative-ion mode under the conditions described below. As shown in Figure 2 and Table 2, eight components were characterized and confirmed based on their retention behaviors and MS data by comparison with [6]. The result proved that the main chemical substances of HLJDT CFP were baicalin (6) and berberine (4), which was coincident with the theory that the CFP might derive from interaction between acidic and basic compounds. To further investigate the possible formation mechanism of HLJDT CFP, baicalin and berberine were selected for the subsequent experiments.

### 2.3. Synthesis of Simulated Precipitation

**Figure 3:**
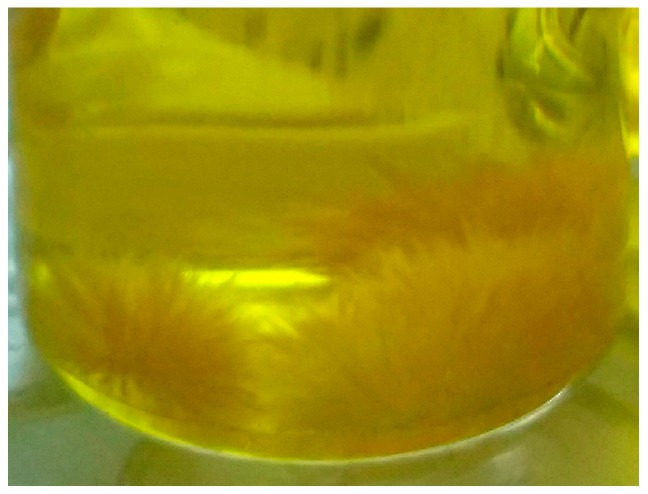
The baicalin–berberine complex in methanol solution.

Simulated precipitation of HLJDT was synthesized by the similar method of the HLJDT CFP. The simulated precipitation was dissolved in dimethyl sulfoxide and crystallized by methanol and acetone. Then the baicalin–berberine complex (Figure 3) was obtained by recrystallization.

### 2.4. Characterization of the Baicalin–Berberine Complex

After melting point determination, we found that the melting process was well-distributed; therefore, the complex could be considered as a homogeneous material. The melting point of the baicalin–berberine complex was measured to be 192.6–193.9 °C, between baicalin (223.7–224.9 °C) and berberine (145.1–146.7 °C).

**Figure 4:**
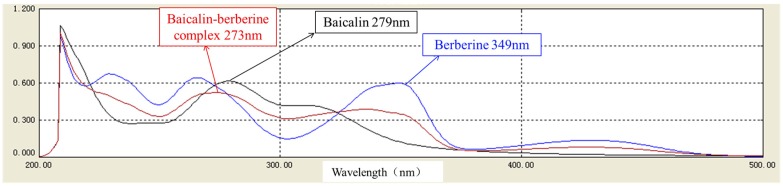
The superimposed UV absorption spectroscopy of baicalin, berberine, and baicalin–berberine complex.

Moreover, the UV absorption spectra of baicalin, berberine, and the complex at 210~500 nm were measured (Figure 4). The UV absorption properties of both baicalin and berberine could be found in the complex; however, the maximum absorption wavelength was changed.

**Figure 5:**
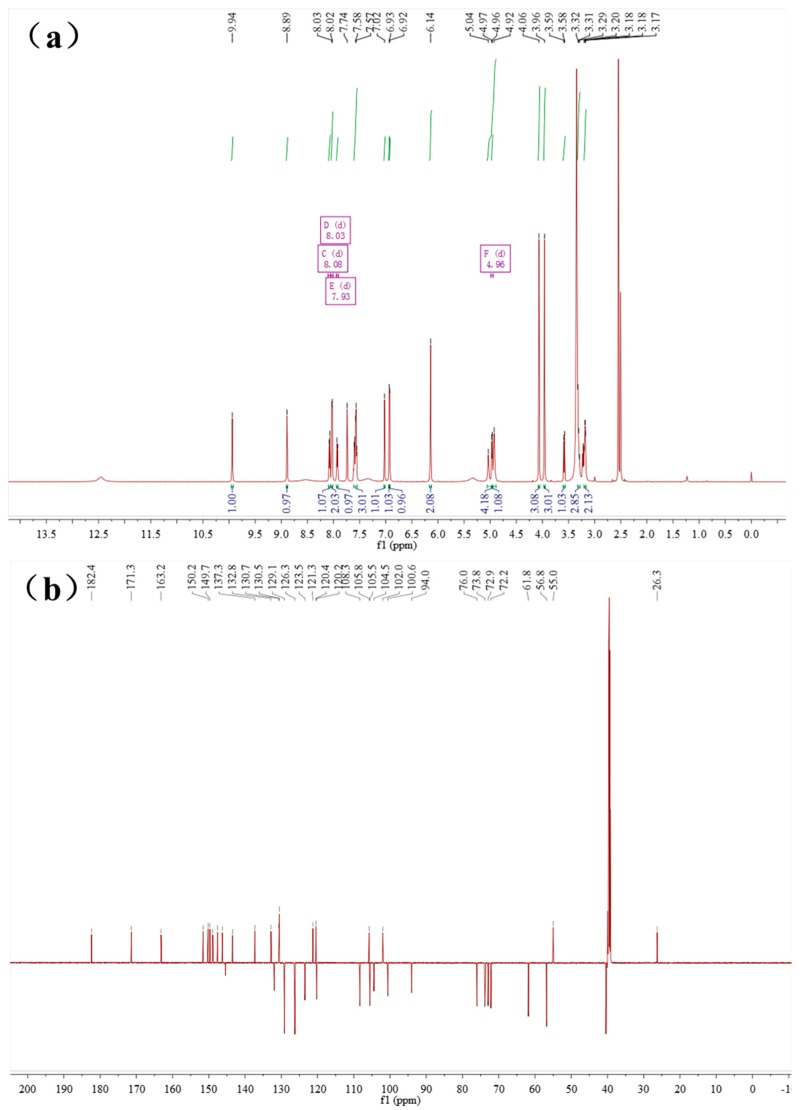
The ^1^H-NMR, ^13^C-NMR of the baicalin–berberine complex. (**a**) ^1^H-NMR of the baicalin–berberine complex; and (**b**) ^13^C-NMR of the baicalin–berberine complex.

Baicalin and berberine were complexed at a ratio of 1:1, which could be found by ^1^H-NMR (Figure 5) integration of the baicalin–berberine complex. In contrast with baicalin, the chemical shift of H and C atoms on the complex’s glucuronide had undergone significant changes by analyzing the ^1^H-NMR and ^13^C-NMR spectra (Table 3). This can revealed by the possible binding site of the baicalin–berberine complex.

**Figure 6:**
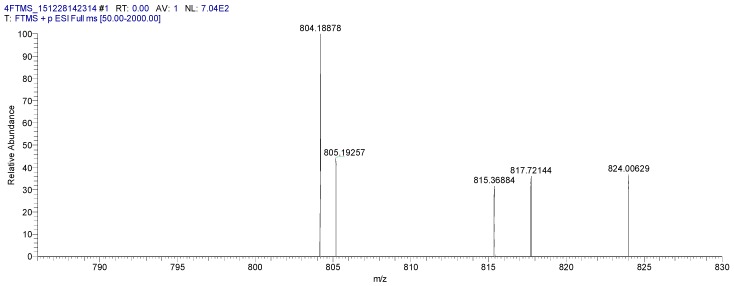
The HR-MS of the baicalin–berberine complex.

The molecular weight 804.18878 [M + Na]^+^ (calcd for C_41_H_35_NNaO_15_ 804.19044) of the baicalin–berberine complex were obtained using HRMS-ESI (Figure 6). The result again proved that baicalin and berberine were complexed at a ratio of 1:1.

### 2.5. Protective Effect of the HLJDT CFP and Baicalin–Berberine Complex on Injured PC12 Cells

**Figure 7:**
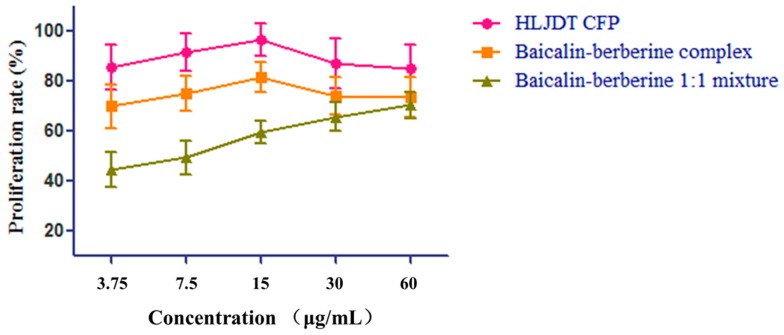
The neuroprotective effect tendency of HLJDT CFP, baicalin–berberine complex, and baicalin–berberine 1:1 mixture. Data are expressed as means ± SD from three separate experiments.

To evaluate the neuroprotective effect of the HLJDT CFP and the baicalin–berberine complex, the two samples and the baicalin–berberine 1:1 mixture were tested on neuronal-like PC12 cells damaged by CoCl_2_. The results (Table 4) showed that both the HLJDT CFP (EC_50_ = 3.35 ± 1.11) and the baicalin–berberine complex (EC_50_ = 5.79 ± 1.67) presented protective effects on injured differentiated PC12 cells. The tendency (Figure 7) of the baicalin–berberine complex is coincident with HLJDT CFP, which can prove their correlation indirectly. The different tendencies of the baicalin–berberine complex and baicalin–berberine 1:1 mixture showed that the complexation between baicalin and berberine can influence the neuroprotective effect.

**Figure 8:**
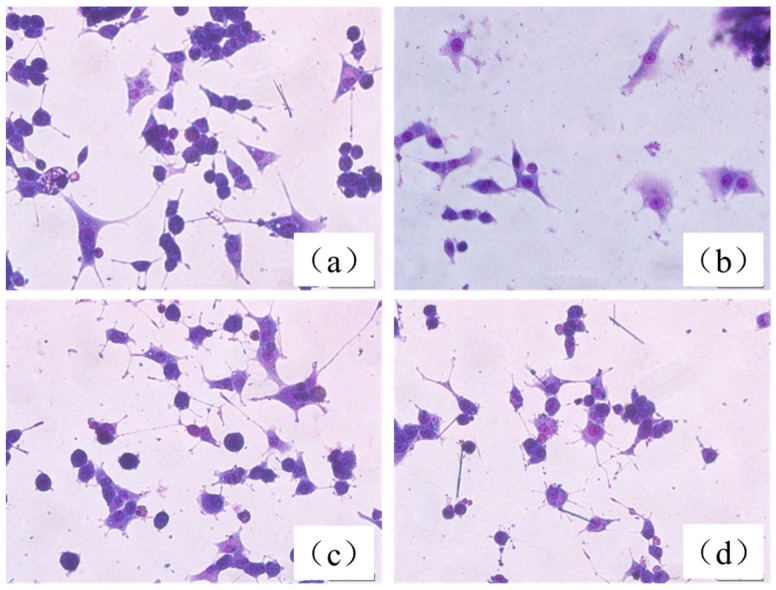
Morphological changes of PC12 cells assessed by Giemsa staining. (**a**) Control group; (**b**) injured by CoCl_2_; (**c**) treated with 15 µg/mL HLJDT CFP then injured by CoCl_2_; and (**d**) treated with 15 µg/mL baicalin–berberine complex then injured by CoCl_2_ (×400).

Under light microscopy, we found that normal, differentiated PC12 cells showed round cell bodies with fine dendritic networks, and the cell edges were intact and clear (Figure 8a). In contrast, incubation of cells with 200 mM of CoCl_2_ for 12 h induced shrinkage of the cell bodies, disappearance of cell reticular formation, and disruption of the dendritic networks (Figure 8b). Pretreatment with 15 µg/mL HLJDT CFP and baicalin–berberine complex dramatically alleviated morphological manifestations of cell damage and led to a pronounced increase in neurite-bearing cells compared to model cells (Figure 8c,d).

**Figure 9:**
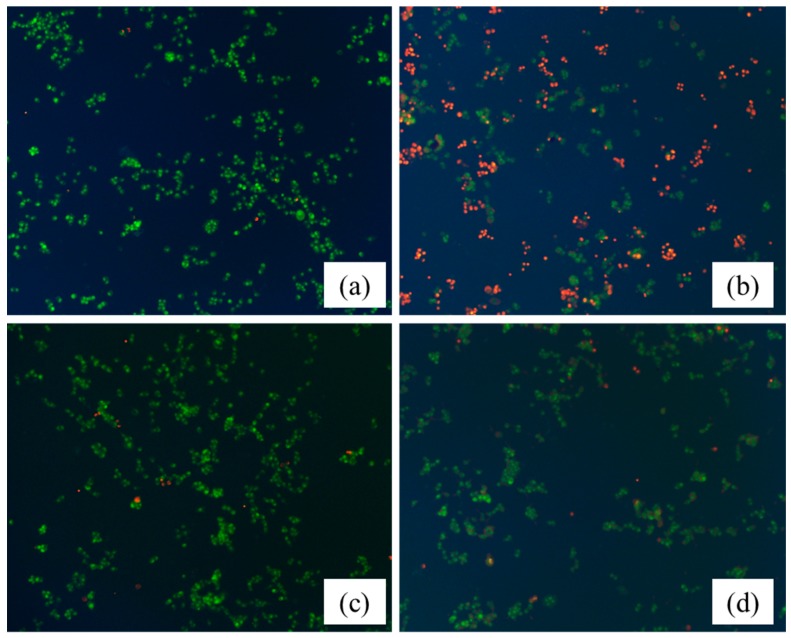
The neuroprotective effect of the HLJDT CFP and baicalin–berberine complex assessed by AO/EB staining. (**a**) Control group; (**b**) injured by CoCl_2_; (**c**) treated with 15 µg/mL HLJDT CFP then injured by CoCl_2_; and (**d**) treated with 15 µg/mL baicalin–berberine complex then injured by CoCl_2_ (×200).

Acridine orange (AO) and ethidium bromide (EB) are fluorescent intercalating DNA dyes. AO can stain nuclear DNA across an intact cell membrane, while EB is only taken by cells that had lost their membrane integrity. Therefore, after stained with AO and EB, live cells will be stained green and regular-sized while late apoptotic and necrotic cells will be stained red. As shown in Figure 9, PC12 cells were treated with 15 µg/mL HLJDT CFP and baicalin–berberine complex for 36 h, and followed by AO/EB staining. Compared with the control group (Figure 9a), the changes on the cell injured by CoCl_2_ (Figure 9b) can be obviously observed. The nuclei, clearly stained as red, suggested cell apoptosis induction by CoCl_2_ on PC12 cells. After treated with the HLJDT CFP (Figure 9c) and the baicalin–berberine complex (Figure 9d), the staining of red decreased in different degrees. The results indicated that both the HLJDT CFP and baicalin–berberine complex prevented the apoptosis induction by CoCl_2_ on PC12 cells.

## 3. Discussion

The HLJDT CFP was obtained by packaging the prescription in a non-woven bag (pore diameter <30 μm) and centrifuging the hot decoction which can avoid the interference of herb residue and separate out the ingredients. The formation rate (2.63% ± 0.20%) indicated that it is certainly beneficial on evaluating medicinal value and raising utilization of the HLJDT CFP. The approximate formation rates of three different batches proved that the formation process of the HLJDT CFP is stable and controllable. A novel LC-ESI-MS^n^ method was developed to identify the constituents of the HLJDT CFP. The eight main components can be used as the quality control indexes of the HLJDT CFP. The results of LC-ESI-MS^n^ indicated that the HLJDT CFP might derive from interaction between acidic and basic compounds. Simulated precipitation of the HLJDT was synthesized and the baicalin–berberine complex was obtained by recrystallization. The melting point determination and UV absorption spectra proved that a new conjugated system unlike baicalin and berberine might generate on the complex. The height of the integral in the ^1^H-NMR spectrum proved that baicalin and berberine were complexed at ratio of 1:1. This can be proved by HR-MS analysis. The chemical shift change of baicalin–berberine complex proved that electron transfer might occur between the carboxyl proton on baicalin glucuronide and the lone pair of electrons on berberine N atoms and then generated a complex precipitate. Knowing the binding site of the baicalin–berberine complex is meaningful for related research of CFP. Both the HLJDT and the baicalin–berberine complex presented protective effects on injured differentiated PC12 cells. The tendency of the baicalin–berberine complex is coincident with the HLJDT CFP，which can prove their correlation indirectly. The different tendencies of the baicalin–berberine complex and the baicalin–berberine 1:1 mixture showed that the complexation between baicalin and berberine can influence the neuroprotective effect. The HLJDT CFP showed better effect than baicalin–berberine complex revealed that there may have other complexes (like baicalin-palmatine complex, Wogonoside-berberine complex, etc.) play effective roles in HLJDT CFP. 

## 4. Materials and Methods

### 4.1. Preparation of HLJDT CFP

The three different batches of HLJDT prescription were purchased from Beijing Tong Ren Tang (Tong Ren Tang Group Co., Beijing, China), a Chinese pharmaceutical company, and the four kinds of medicinal herb were identified. The HLJDT prescription was packaged in a non-woven bag (pore diameter <30 μm) and heated in a reflux with 8 times the amount of water for 30 min. Then the HLJDT water decoction was centrifuged at 4000 r/min for 15 min while still hot. The supernatant solution was removed and the HLJDT CFP was vacuum dried at 30 °C. The precipitation rate (%) was calculated in the following equation:

Precipitation rate% = (HLJDT CFP weight/HLJDT prescription weight) × 100%
(1)

### 4.2. LC-MS^n^ Analysis of the Constituents of HLJDT CFP

The LC-ESI-MS^n^ analysis was performed with an Agilent 1100 LC system with an LC/MSD Trap XCT Plus mass spectrometer (Agilent Technologies, Santa Clara, CA, USA). A TC-C18 (4.6 mm × 250 mm, 5 μm, Agilent) column was used for analysis. The column temperature was kept at 30 °C. The mobile phases consisted of methanol (A) and water containing 1% formic acid (B). The following gradient condition was used: 0–10 min, 10% A; 10–12 min, 10%–30% A; 12–30 min, 30%–35% A; 30–35 min, 35%–40% A; 35–40 min, 40%–55% A; 40–50 min, 55%–66% A; and 50–60 min, 66%–100% A. The mobile phase flow rate was 0.8 mL/min and the sample injection volume was 10 μL. The detected wavelength was 254 nm. Mass spectra were acquired in both positive and negative ion modes with an ESI source in the range of *m*/*z* 100 to 1000. The ESI-MS conditions were: the nebulizer pressure at 45 psi and nitrogen as the drying gas at a flow rate of 10 L/min with a temperature of 350 °C. The capillary voltage was set at 3500 V. Data were acquired by use of Agilent Chemstation software (Agilent Technologies).

### 4.3. Synthesis of Simulated Precipitation

Baicalin and berberine were dissolved in boiling water and stirred for 0.5 h, then the colloid precipitation was collected by suction filtration using a Hirsch funnel and dried it at 30 °C. The baicalin–berberine complex was obtained by dissolving the simulated precipitation in dimethyl sulfoxide and crystallizing by methanol and acetone.

### 4.4. Characterization of the Baicalin–Berberine Complex

The melting point was determined by a Tektronix X-5 microscopic melting point detector (Beijing Tektronix Department of Micron Technology Inc., Beijing, China). The absorption wavelength was measured by a U-2000 UV-visible spectrophotometer (Hitachi Ltd., Tokyo, Japan). ^1^H-NMR and ^13^C-NMR assays were recorded on an AVANCE III 600 NMR spectrometer (BRUKER Corporation, Billerica, MA, USA) with tetramethylsilane (TMS) as an internal standard and chemical shifts are reported in δ (ppm). HR-MS were obtained by using Synapt G2 high-resolution mass spectrometer (Waters Corporation, Milford, MA, USA).

**Figure 10:**
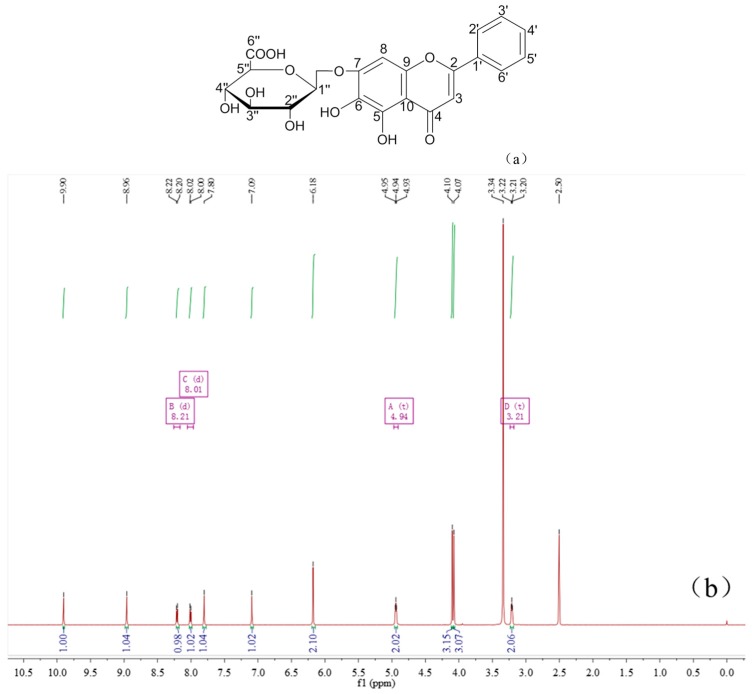

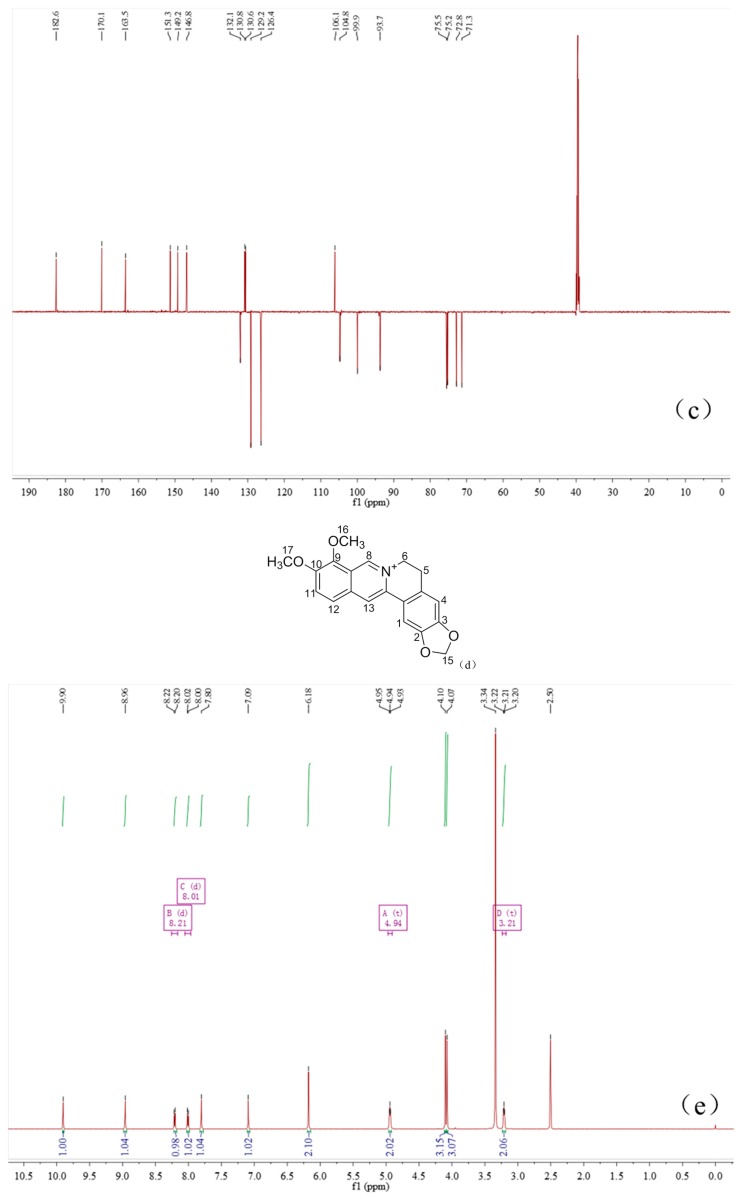

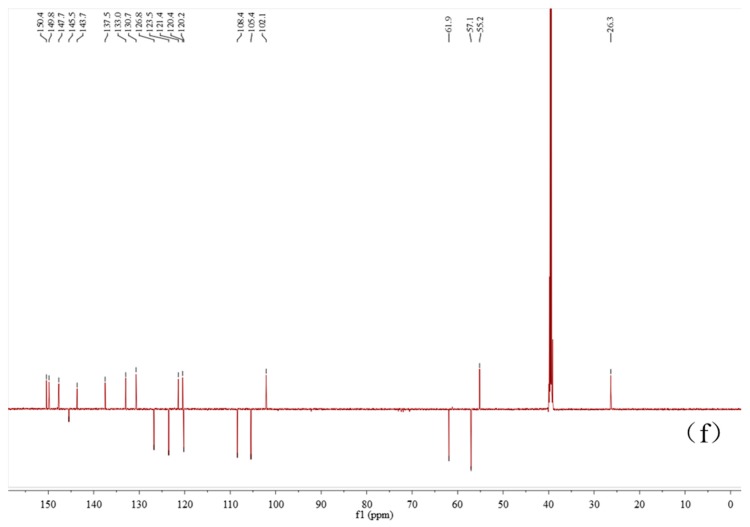
The ^1^H-NMR, ^13^C-NMR and structure of baicalin and berberine. (**a**) Structure of baicalin; (**b**) ^1^H-NMR of baicalin; (**c**) ^13^C-NMR of baicalin; (**d**) structure of berberine; (**e**) ^1^H-NMR of berberine; and (**f**) ^13^C-NMR of berberine.

The ^1^H-NMR and ^13^C-NMR of Baicalin–berberine complex was analyzed by referring the ^1^H-NMR and ^13^C-NMR of baicalin and berberine (Figure 10).

The baicalin–berberine Complex, pale yellow crystal, m.p.: 192.6–193.9 °C. ^1^H-NMR (600 MHz, DMSO-*d*_6_) (ppm): 9.94 (s, 1H, berberine 8-H), 8.89 (s, 1H, berberine 13-H), 8.08 (d, *J* = 9.0 Hz, 1H, berberine 11-H), 8.03 (d, *J* = 7.4 Hz, 1H, baicalin 2’, 6’-H), 7.93 (d, *J* = 9.0 Hz, 1H, berberine 12-H), 7.74 (s, 1H, berberine 1-H), 7.60-7.55 (m, 3H, baicalin 3’, 4’, 5’-H), 7.02 (s, 1H, berberine 4-H), 6.93 (s, 1H, baicalin 8-H), 6.92 (s, 1H, baicalin 3-H), 6.14 (s, 2H, berberine 15-CH_2_-), 5.04–4.92 (m, 3H, baicalin 2”, 3”, 4”-OH), 4.96 (d, *J* = 6.8 Hz, 1H, baicalin 1”-H), 4.06 (s, 3H, berberine 16-OCH_3_), 3.96 (s, 3H, berberine 17-OCH_3_), 3.58 (d, *J* = 9.8 Hz, 1H, baicalin 5”-H), 3.32–3.29 (m, 3H, baicalin 2”, 3”, 4”-H,), 3.18 (t, *J* = 6.0 Hz, 2H, berberine 5-CH_2_-). ^13^C-NMR (150 MHz, DMSO-*d*_6_) (ppm): 182.4 (baicalin 4-C), 171.3 (baicalin 6”-C), 163.2 (baicalin 2-C), 151.6 (baicalin 7-C), 150.2 (berberine 3-C), 149.7 (berberine 10-C), 148.9 (baicalin 5-C), 147.5 (berberine 2-C), 146.3 (baicalin 9-C), 145.4 (berberine 8-C), 143.5 (berberine 9-C), 137.3 (berberine 13a-C), 132.8 (berberine 12a-C), 132.0 (baicalin 6-C), 130.7 (baicalin 1’-C), 130.5 (baicalin 4’-C), 129.1(baicalin 3’, 5’-C), 126.3(baicalin 2’, 6’-C), 123.5 (berberine 11-C), 121.3 (berberine 8a-C), 120.4 (berberine 13-C), 120.2 (berberine 1a-C), 108.3 (berberine 4-C), 105.8 (baicalin 10-C), 105.5 (berberine 1-C), 104.5 (baicalin 3-C), 102.0 (berberine 15-C), 100.6 (baicalin 1”-C), 94.0 (baicalin 8-C), 76.0 (baicalin 5”-C), 73.8 (baicalin 3”-C), 72.9 (baicalin 2”-C), 72.2 (baicalin 4”-C), 61.8, 56.8 (berberine 16, 17-C), 55.0 (berberine 6-C), 26.3 (berberine 5-C).

### 4.5. Protective Effect of HLJDT CFP and Baicalin–Berberine Complex on Injured PC12 Cells

PC12 cells were cultured in RPMI 1640 medium supplemented with 5 % (*v*/*v*) fetal bovine serum, 10% (*v*/*v*) horse serum and 100 U/mL penicillin-streptomycin (Thermo Technologies, New York, NY, USA) at 37 °C in a humidified atmosphere of 5% CO_2_. When cells achieved the desired density of >80% confluency, the original medium was removed and cells were cultured with the serum-free medium for 14 h. Then the cells were suspended in 1640 medium supplemented with 10% (*v*/*v*) fetal bovine serum, and seeded into poly-l-lysine-coated 96-well culture plates at 7 × 10^3^ cells/well, differentiated and treated with 50 ng/mL nerve growth factor (NGF) for 48 h. After these, the differentiated PC12 cells were pretreated with various concentrations (60, 30, 15, 7.5, 3.75 µg/mL) of samples for 36 h. All measurements were performed after the cells were induced by CoCl_2_ (final concentration, 200 mM) for 12 h. Control differentiated cells were not treated. CoCl_2_ was dissolved in RPMI 1640 medium. Samples were dissolved in DMSO. The final concentration of DMSO was less than 0.1% (*v*/*v*). After MTT solution (20 µL, 5 mg/mL) was added to each well, the plate was incubated for a further 4 h at 37 °C. The supernatant was removed carefully by pipetting from wells without disturbing the attached cells and formazan crystals were solubilized by adding 200 µL of DMSO to each well and shaken for 15 min. The absorbance at 490 nm was measured with a BIORAD 550 spectrophotometer (Bio-Rad, Berkeley, CA, USA). The proliferation rates of damaged PC12 cells were calculated by the formula [OD490 (Sample) − OD490 (CoCl_2_)]/[OD490 (NGF) − OD490 (CoCl_2_)] × 100%.

Giemsa staining and AO/EB staining were performed according to our previous study with minor modifications [26,27]. Morphological changes were examined using an Olympus IX71 inverted microscope (Olympus, Tokyo, Japan) with 400× actual magnification. The fluorescence was observed with 200× actual magnification.

## 5. Conclusions 

In summary, the main chemical substances of HLJDT CFP were identified by a novel LC-MS^n^ method. To investigate the formation mechanism of HLJDT CFP, baicalin and berberine were selected to synthesize simulated precipitation and a baicalin–berberine complex was obtained. The melting point and UV absorption of the baicalin–berberine complex were different from the raw material. In addition, ^1^H-NMR integral and HR-MS can validate that the binding ratio was 1:1. Compared with baicalin, the chemical shifts of H and C on glucuronide have undergone significant changes by ^1^H-, ^13^C-NMR, which proved that electron transfer occurred between the carboxylic proton and the lone pair of electrons on the N atom. Both HLJDT CFP and the baicalin–berberine complex showed protective effects against cobalt chloride-induced neurotoxicity in differentiated PC12 cells. This kind of method may be a revelation on studying the material foundation of CFP in Chinese prescriptions. 

## Figures and Tables

**Table 1:** The HLJDT CFP formation rate from three different batches.

Batch	Coptidis Rhizoma	Scutellariae Radix	Phellodendri Cortex	Gardeniae Fructus	Total Weight	Precipitation Weight	Precipitation Rate
1	9.02 g	6.00 g	6.01 g	9.02 g	30.05 g	0.73 g	2.44%
2	9.01 g	5.99 g	6.01 g	9.01 g	30.02 g	0.85 g	2.84%
3	9.00 g	6.00 g	5.99 g	9.00 g	29.99 g	0.78 g	2.61%
average					30.02 ± 0.03 g	0.79 ± 0.06 g	2.63% ± 0.20%

**Table 2:** ESI-MS^n^ ions of the identified compounds.

Peak	Compounds	*t*_R_ (min)	MS (*m/z*)	MS^n^ (*m*/*z*)
1	Geniposide	21.36	386.9 [M − H]^−^	224.8 [M − H − C_6_H_10_O_5_]^−^ 123.0 [M − H − C_6_H_10_O_5_ − C_4_H_6_O_3_]^−^
2	Coptisine	24.15	320.0 [M]^+^	292.0 [M − C_2_H_4_]^+^
3	Epiberberine	24.86	336.1 [M]^+^	320.1 [M − CH_4_]^+^ 292.1 [M − CH_4_ − C_2_H_4_]^+^
4	Berberine	28.17	336.1 [M]^+^	320.1 [M − CH_4_]^+^ 292.1 [M − CH_4_ − C_2_H_4_]^+^
5	Palmatine	29.60	352.1 [M]^+^	336.1 [M − CH_4_]^+^ 308.1 [M − CH_4_ − C_2_H_4_]^+^
6	Baicalin	41.76	447.1 [M + H]^+^	271.0 [M + H − glu^a 1^]^+^
7	Oroxylin A A-7-*O*-glucuronide	44.46	458.9 [M − H]^−^	282.8 [M − H − glu^a^]^−^
8	Wogonoside	45.10	458.9 [M − H]^−^	282.8 [M − H − glu^a^]^−^

^1^ glu^a^: gluconic acid.

**Table 3:** The ^1^H-NMR, ^13^C-NMR chemical shift change of the baicalin–berberine complex, contrasted with baicalin.

Baicalin (δ ppm)	Baicalin–Berberine Complex (δ ppm)	Difference (ppm)
2’’, 3’’, 4’’-OH, 5.52–5.31	5.04–4.92	0.39–0.48
1’’-H, 5.26	4.96	0.30
5’’-H, 4.08	3.58	0.50
2’’, 3’’, 4’’-H, 3.47–3.42	3.32–3.29	0.13–0.15
6”-C, 170.1	171.3	1.2
1”-C, 99.9	100.6	0.7
5”-C, 75.5	76.0	0.5
3”-C, 75.2	73.8	1.4
4”-C, 71.3	72.2	0.9

**Table 4:** The protective effect of HLJDT CFP, baicalin–berberine complex, and baicalin–berberine 1:1 mixture on injured PC12 cells. Data are expressed as means ± SD from three separate experiments.

Samples	Proliferation Rate (%)					EC_50_ (μg/mL)
	60 μg/mL	30 μg/mL	15 μg/mL	7.5 μg/mL	3.75 μg/mL	
HLJDT CFP	85.08 ± 9.72	86.98 ± 10.03	96.79 ± 6.52	91.53 ± 7.58	85.58 ± 9.03	3.35 ± 1.11
Baicalin–Berberine complex	73.62 ± 7.96	74.17 ± 7.46	81.48 ± 6.08	75.10 ± 6.94	70.05 ± 8.72	5.79 ± 1.67
Baicalin–Berberine 1:1 mixture	70.45 ± 5.36	65.83 ± 5.85	59.43 ± 4.43	49.38 ± 6.57	44.57 ± 6.84	11.1 ± 2.49
